# American Dental Association

**Published:** 1884-12

**Authors:** Chas. Mayr


### AMERICAN DENTAL ASSOCIATION.

### TWENTY-FOURTH ANNUAL MEETING, AT SARATOGA.

REPORTED FOR THE INDEPENDENT PRACTITIONER BY PROF. CHAS. MAYR.

(Continued from page 631.)

Section VII was called, Physiology and Etiology. The report was presented by the Chairman, Dr. W. C. Barrett.*

DISCUSSION.

*Prof. Chas. Mayr*—Almost all the differences in the several views about caries seem to come from an indefiniteness of what and where the caries really is. If a patient comes to you with an aching tooth, is the ache the caries? Certainly not. Or the tooth? Again not. Or the plug you dig out? Not at all; but while you began to dig the patient first said nothing, then he became nervous and complaining, as you reached a certain point, which irritability again disappeared when you came into healthy dentine. The caries comprises, therefore, only that small diseased layer, probably not over $\frac{1}{100}$ of an inch thick, that was sensitive; outside we have the worthless plug or corpse of the tooth substance; inside, the healthy tooth. Dr. Miller seems to have mostly examined the corpse and produced the corpse, but not enough of that extremely small border-line. In an abscess the pus is not the seat of the disease, nor the inflamed tissue underneath, but that small layer of tissue where the physiological—or if some fogies like the word better, pathological—processes are going on, and the study of this layer will enlighten us about the abscess, but neither the pus nor the inflamed tissue underneath will tell us much.

*Dr. Atkinson*—What is a process? It is a-going on of something. What is a thing? A thing is a think. Pathology is only perverted physiology. The function of building a tooth is its evolution; the decay, decadence, its downfall. The typal order of the enamel is a modification of that of the dentine; the enamel is really a living tissue, as shown by the presence of protoplasmic strings between the enamel rods. The bacteria are not found in the basis substance, but in the tubuli of the dentine, going towards the pulp.

* The paper is omitted for want of space.—EDITOR.

*Dr. Spaulding*—I have long held the opinion that there is no pathological science except it be preceded by physiological science; that there is no pathology *per se*, and that there are no such things as pathological functions, processes and growths. They are physiological functions, processes and growths, out of place. It is the inversion of true physiological forces that produces the exhibition of pathological growth.

*Dr. Barrett*—We cannot speak of the power of a bacterium as that of a raging animal, boring into the teeth for the purpose of devouring living tissue. That is not the character of the fungi of caries. They are of a vegetable nature. Putrefactive and fermentative conditions are almost the converse of each other. Putrefaction does not exist at the same time with fermentation. From my own standpoint, the rationale of decay is about like this: First, there will be decalcification of the exterior portion of the enamel; thus the dentine is reached, and at perhaps a single point the lime salts are removed. The decalcified substance becomes a spongy mass that takes up within its pores saccharine fluids from the mouth. These are favorable to the development of fermentive fungi. In the course of their existence they produce an acid which Dr. Miller proves to be lactic acid, which continues the work of decalcification, and so *ad infinitum.*

*Dr. Abbott*—Dr. Barrett said we ought to deal with actualities, and not with ghosts; what we have been able to discern with the naked eye or the strongest power of the microscope. Does this enable us positively to say that there is nothing beyond ? Dr. Miller seems to neglect the fact that the tooth has twenty-five to thirty per cent. of organic material, which makes of it a living organ.

The discussion was brought to a close by the arrival of the hour set apart for the organization of the sections, and this business occupied the society until the time for adjournment.

WEDNESDAY EVENING SESSION.

By special vote of the society the evening session was occupied by Dr. J. L. Williams, of New Haven, Conn., who read a paper upon "The Origin of Enamel and the Nature of the Odontoblast Cells." It was illustrated by a great number of very beautiful preparations, the images of which were projected upon a screen by means of the stereopticon. Owing to the necessary darkening of the hall, as well as to the absorbing beauty of the preparations, which continually distracted the reporter's attention, the notes of the paper are not as full and perfect as they might otherwise have been.

The speaker commenced by criticising the expressed views of Dr. Garretson, Dr. Cryer and others, and stated that he would demonstrate they were based upon faulty preparations and imperfect knowledge.

He had presented this matter at the last meeting of the New England Dental Society, and it was then privately remarked that it was of little importance whether or not his own theory was the correct one. He differed from them, for it was impossible for one to undertake, with any hope of success, such a work as the joining together of parts of broken teeth, without a definite knowledge of the nature of the process. Besides, as professional men, it is our bounden duty to obtain all the knowledge possible of the tissues upon which we operate.

It is of the greatest importance that the student should be correctly taught concerning this subject. The purpose of this paper was to point out the most important fallacies still existing in the minds of dentists concerning the structure of the teeth. For that purpose he would consider what is stated about the development of the papilla in the pulp, and show that there is an enamel organ which has a definite structure, and that the formation of this enamel organ takes place from the dentine outward. He would show that the odontoblasts are ganglionic elements, sending branches into the dentine and the pulp. To answer the question why this was not established before, he would say that it is very difficult to obtain specimens that show the natural relations of the dental tissues to each other and to the surrounding tissues without breaking the continuity of the structure. A great number of microscopic slides were shown, most of them photographic; a few, diagramatic.

*First slide*—From a professional dealer in microscopical specimens. In this you may see the appearance of what Dr. Garretson calls the *utricula reflexa*. All the delicate structure, the basement membrane, and the enamel organ are destroyed; this slide has some historic value, just as the implements of the stone age tell us of the progress that has been made.

*Second slide*—This is in contrast with the foregoing. The old views were derived from balsam-mounted specimens, which define less distinctly than those mounted in glycerine. This slide shows the first differentiation that marks the beginning of the tissues; it is perfect in every way, except at one point. The groove of which Dr. Garretson speaks is filled out with the enamel organ, which is destroyed very easily by careless preparation. The former logic in support of the theory about the formation of the enamel ran thus: The enamel is an epidermoid structure; all epidermoid structures are formed in the same manner—ergo, the enamel must be formed in the same manner. This is only a trick of logic, not supported by any evidence. A vast amount of ignorance may conceal itself behind a general physical law. A little definite knowledge is better than generalizations, which may sometimes mean something, but generally do not. How a tissue which never grows after it has been destroyed can be compared to one which is constantly thrown off and replaced by a new one, is beyond comprehension.

*Third slide*—Tinted a very little with carmine, beautifully shows the origin of the dental tubuli and their ends.

*Fourth slide*—Shows a permanent tooth, with the pulp removed and the dentine cells in some places intentionally forced away from the enamel.

*Fifth slide*—Shows a cut through a temporary tooth, a little to one side of the center, cutting through the completely formed enamel with its hexagonally divided appearance.

*Sixth slide*—Shows something like crotchet work. It is possible that the enamel may be formed by intercellular deposits; of such formations we have analogies; the placoid scales are formed in the same manner; they have spinous projections that have an enamel layer which is deposited as a cuticular deposit of the epidermis. This crotchet-like looking tissue is from the embryonic shark; it looks like an embryonic tooth.

*Seventh slide*—From Balfour's embryology, shows the formation of placoid scales in a placoid fish, identical with the development of the teeth.

*Eighth slide*—Shows the cord which is drawn down at the first folding of the dental cyst, and which develops from the columnar layer of epithelium. It is already broken from its union with the upper layer, and the tooth cyst is isolated thereby; this specimen shows also the development of a beautiful reticulum of the enamel organ; I have not determined whether it is developed by direct reformation of cells, or by a breaking down of embryonal tissue. After the completion of the enamel the organ is exhausted, yet it remains on the lower side of the tooth until its work is also done there, after which it disappears.

*Ninth slide*—Shows an additional proof of the existence of the enamel organ; the enamel-forming cells and the reticulum are there, but the stellate cells, seen in the former specimen, are no more. On the top of the tooth the enamel organ has disappeared, and the calcification is complete. At the sides it is not complete, and therefore it still exists there. The pericementum makes its appearance, and thus a connection of the tooth with the external world is established.

*Tenth slide*—You remember that on a previous slide, No. 8, at the lower end of the enamel cap, there was shown a columnar layer of cells which came, in the development of the cord, from the columnar layer of epithelium above. This is a highly magnified view of this point at a later stage. The columnar layer has disappeared, and the space is occupied by indefinite embryonal tissue, or indifferent corpuscles. The reticulum of the enamel pulp, shown here, is very beautiful. The embryonal corpuscles forming the lower part of the indifferent tissue are developed from the ameloblasts, or enamel-forming cells.

*Eleventh slide*—Shows a somewhat advanced tooth germ; the ameloblasts are above the flattened layer; above it is also visible the reticulum of the enamel organ.

*Twelfth slide*—Shows the ameloblasts in the form of biscuits; the analysis proves that this portion is highly impregnated with lime salts. I call your attention to the odontoblasts. You know that Dr. Heitzman and others have represented them as round corpuscles, between which the dental fibrillæ pass. I have satisfied myself that this has been an erroneous view, but it was not until within a short time that I have succeeded in cutting a section so thin that only one layer of odontoblasts was obtained. I can show you photographs in which the dental pulp and the enamel pulp are in perfect contact, and others where the first faint traces of dentine and enamel appear. (The photographs were here exhibited.)

This specimen I exhibited to Drs. Heitzman, Bödecker, and Atkinson; Prof. Heitzman only glanced at it, when he turned to Dr. Bödecker with frankness and said, "It is evident we have made a mistake;" subsequently, he thought he could detect delicate fibres passing between the fibrils.

*Thirteenth slide*—You see the prolongation of the odontoblasts into the tubuli; one point shows two fibrillæ originating from the odontoblasts. I discovered that sometimes three or more fibrillæ are projecting.

*Fourteenth slide*—I have often been asked how I explained the formation of dentine. Do these cells perform a double function? are they the elements of sensation as well as of the formation of dentine? We often see a multiplicity of functions performed by embryonal cells. The odontoblasts are essentially neural matter, and are probably also the secretive elements of the dentine. Some species of molluscs show similar cells in the digestive tract, and they are exhibited in this photographic slide, thus proving their multiplicity of function. I trust that this addition will prove of benefit to all. I have sometimes spoken a little sharply, because I would not like to see the results of the study of years set aside by a hasty examination of inferior specimens and sections.

At the close of the very instructive and interesting address of Dr. Williams, Dr. J. H. McKellops asked the suspension of the order of business that an announcement might be made. Leave being granted, he called upon Dr. W. C. Barrett, who aroused the sympathy of every member by relating the particulars of a very painful accident that had just occurred. The little, motherless boy of Dr. W. R. Clifton, of Texas, was, in company with his nurse, crossing the street, when a carriage, driven at a reckless speed, ran over him, crushing the skull and breaking a number of bones. The sympathy of the Association was tendered Dr. Clifton, and the executive committee was, by vote, directed to engage physicians and nurses, and to take measures to bring the criminally reckless driver to justice.

*Dr. Atkinson*—Desired to express his obligation to the writer of the paper, and had commenced a consideration of some of its points, when he was interrupted by Dr. C. W. Spalding, who said that he had been so shocked by the relation of the terrible accident that he was in no mood for a discussion, and he thought others were in the same state. He therefore asked the speaker in possession of the floor to give way for a motion of adjournment.

*Dr. Atkinson*—Said that he was astonished at the puerility of the excuse for adjournment. We were never more clearly in the way of right than when we were pursuing the path of duty and earnestly laboring at the business directly before us. He declined to give way, and continued his remarks, but the sentiment of the meeting was evidently against him, and the members left in such numbers that an adjournment for want of a quorum was soon a necessity. The painful accident seriously interfered with the consideration of the paper.

